# Editorial: The mucosal barrier to infection

**DOI:** 10.3389/fcimb.2023.1141131

**Published:** 2023-01-30

**Authors:** Yong Hua Sheng, Li Zhang

**Affiliations:** ^1^ Immunopathology Group, Mater Research Institute, The University of Queensland, Translational Research Institute, Brisbane, QLD, Australia; ^2^ School of Biotechnology and Biomolecular Sciences, University of New South Wales, Sydney, NSW, Australia

**Keywords:** mucosal, epithelia cells, microbiota, pathogens, host defense

The gastrointestinal tract (GIT) and respiratory tract (RIT) have large areas exposed to the external environment, requiring sophisticated defence strategies to protect against infectious agents and maintain mucosal homeostasis in the presence of microbiota containing trillions of microbes. The mucosal barrier, formed by epithelial cells and mucus, prevents microorganisms and noxious substances from reaching the epithelium. In addition to forming a cellular barrier for protection of the underlying tissues, the epithelial cells also send signals to immune cells to initiate immune responses on detection of harmful microbes. A greater understanding of interactions between the mucosal barrier and microbes including both pathogens and commensal microbes will provide insights into the establishment of novel strategies in the prevention and reduction of mucosal infection and inflammatory diseases.

A diverse community of large numbers of commensal bacterial species inhabit mucosal surfaces in humans. In healthy individuals, the components of commensal bacteria and their metabolites regulate the function of epithelial cells and induce protective immune responses which can prevent the pathogens and their products from contacting the host epithelium (Round and Mazmanian, 2009; Zheng et al., 2020). On the other hand, the epithelial cells and the mucosal immune system play a role in shaping the composition of the microbiota (Liu et al., 2018). Enteric pathogens disturb the diversity of gut microbiota and affect the physiology of epithelial cells. However, the interactions between gut microbiota and intestinal epithelium under infection are largely not clear. In this Research Topic, Zhou et al. provided updated information by reviewing studies investigating the crosstalk between the gut microbiota and epithelial cells under physiological and infectious conditions.

The mucosal epithelium is lined by mucus. Mucus is formed from heavily glycosylated proteins, namely mucins, which are either secreted or membrane associated. The mucus, together with embedded antimicrobial peptides and IgA secreted by epithelial and immune cells, plays a critical role in mucosal homeostasis. The mucus interacts with microbiota and limits their contact with host epithelium. However, many enteric pathogens have evolved strategies to infect the host by destroying or avoiding the mucus barrier. The review by Sheng and Hasnain in this Research Topic provided evidence for the mucins at the mucosal surfaces as a key part of innate immunity. Mucins can dictate the composition of the microbiota, provide essential physical and chemical scaffolds within the mucosal barrier, and are closely interlinked with the adaptive immune system. They emphasised the dynamic function of the mucins and mucus during infection, how this mucosal barrier is regulated and how pathogens have evolved mechanisms to evade this defence system.

In healthy individuals, microbes constituting the gut microbiota regulate the function of the intestinal epithelial cells and mucosal immune system, however they do not induce inflammation and are therefore referred to as commensal microbes. In individuals with impaired epithelial barrier and inflammatory conditions such as inflammatory bowel disease (IBD), commensal gut microbes participate in or even become the main player driving intestinal inflammation (Zhang et al., 2022). Upon intestinal barrier damage, an individual’s gut microbial community determines the severity of inflammation induced by commensal microbes (Roy et al., 2017). Consequently, modulation of the gut microbiota has been suggested as a therapeutic strategy for IBD. In the research article from Zhu et al., a novel probiotic cocktail was formed and their role in alleviation of intestinal inflammation was examined in comparison with fecal microbiota transplantation and use of anti-inflammatory drug 5-aminosalicylic acid in a dextran sulfate sodium (DSS) induced colitis model. Zhu et al. showed that the novel probiotic cocktail they have developed performed better than fecal microbiota transplantation from a healthy mouse donor and 5-aminosalicylic acid in decreasing intestinal inflammation. Furthermore, they showed that the probiotic cocktail significantly increased the junction protein JAM-1 expression in the colon. The authors attributed these beneficial effects of their probiotic cocktail to the increase of anti-inflammatory bacterial species and decrease of pro-inflammatory bacterial species in the murine gut microbiota. These interesting findings provided further evidence that the composition of gut microbiota affects the severity of intestinal inflammation in animal hosts with a damaged intestinal barrier. Future studies in humans are required to further assess whether this probiotic cocktail is clinically beneficial to patients with IBD.

The mucosal surfaces face threats from different types of pathogens and infection of one pathogen may impact on the host response to another. *Pseudomonas aeruginosa* is a Gram negative opportunistic human bacterial pathogen, which often causes chronic infections in patients with cystic fibrosis (CF). It is unclear how the chronic infection of *P. aeruginosa* impacts the responses from patients with CF to viral infections; this was investigated by Endres et al. in a study published in this Research Topic. Using differentiated primary bronchial epithelial cells, Endres et al. showed that co-infection with a non-mucoid *P. aeruginosa* isolate increased bronchial epithelial production of IL-1ß but significantly decreased the production of IL-6 induced by human rhinovirus (HRV). They also observed that co-infection of the non-mucoid *P. aeruginosa* isolate and HRV had a higher adverse impact on bronchial cell function, demonstrated by lower mRNA levels of Forkhead box J1 (FOXJ1) and cilia Apical Structure Protein (SNTN); both are markers for epithelial cells differentiation to ciliated cells. Increased epithelial permeability was observed in co-infections than single infections. These interesting findings provide evidence that chronic mucosal infection and inflammation alter innate immune responses to other pathogens.

Mucins are not only the critical component of the mucus covering the mucosal epithelial cells, but are also present in human saliva, which act as decoys to oral microbial binding to hinder bacteria from binding host surfaces in addition to providing lubrication and hydration (Lindén et al., 2009). MUC5B and MUC7 are the predominant mucins in saliva. Several *Streptococcus* species such as *Streptococcus oralis* in the human oral cavity are opportunistic pathogens and can enter the bloodstream and cause bacteremia and infective endocarditis. Chahal et al. investigated the mechanisms by which *S. oralis* binds to MUC5B and MUC7. They showed that *S. oralis* bound both MUC5B and MUC7 and identified the respective receptors which included Leb, LNT and SLex like glycans on the two mucins and that the mechanisms of binding MUC5B and MUC7 are different. Effective binding of *S. oralis* to salivary mucins required Sortase A-dependent cell wall anchored surface protein(s), including AsaA, and another unidentified LPXTG-containing adhesin(s). These findings suggested that further studies are needed to reveal of these adhesins.

In conclusion, this Research Topic consisted of both review and original research articles contributed by 35 authors, which provides novel information and comprehensive reviews on the interactions between mucosal epithelial cells and mucus with microbiota and pathogens.

## Author contributions

YS provided the suggestions on drafts of the manuscript and LZ wrote the manuscript. All authors contributed to the article and approved the submitted version.
